# Septic shock due to *Aeromonas hydrophila* bacteremia in a patient with alcoholic liver cirrhosis: a case report

**DOI:** 10.1186/1752-1947-8-402

**Published:** 2014-12-03

**Authors:** Tetsuya Yumoto, Shingo Ichiba, Nao Umei, Sunao Morisada, Kohei Tsukahara, Keiji Sato, Toyomu Ugawa, Yoshihito Ujike

**Affiliations:** Advanced Emergency and Critical Care Medical Center, Okayama University Hospital, 2-5-1 Kita-ku, Shikata-cho, Okayama-shi, Okayama, 700-8558 Japan

**Keywords:** *Aeromonas hydrophila*, Liver cirrhosis, Septic shock

## Abstract

**Introduction:**

*Aeromonas hydrophila* sometimes causes bacteremia, which can be fatal in compromised patients, such as those with liver cirrhosis. We present a case of septic shock due to *Aeromonas hydrophila* bacteremia in a patient with liver cirrhosis, which was successfully treated with rapid resuscitation and critical care.

**Case presentation:**

A 71-year-old Japanese man with liver cirrhosis was transported to our emergency center by ambulance after presenting with gait difficulties and fever. On arrival, he exhibited shock and severe lactic acidosis, which was suggestive of sepsis, and was immediately resuscitated and administered empiric antibiotic therapy. He also displayed catecholamine-resistant hypotension, which was successfully treated with critical care including supportive therapies, such as polymyxin B hemoperfusion and cytokine-absorbing hemofiltration. *Aeromonas hydrophila* was detected in his initial blood cultures.

**Conclusions:**

*Aeromonas* septicemia should be considered in patients with alcoholic liver cirrhosis who have profound shock. In addition to goal-directed therapy and the prompt administration of empiric antibiotic therapy, aggressive critical care involving multiple supportive therapies can save such patients.

## Introduction

*Aeromonas* species cause a wide spectrum of diseases such as gastroenteritis, wound infections, and septicemia. Diarrheal disease is the most common type of condition associated with *Aeromonas* infection. Penetrating or abrasive injuries that occur in an aquatic environment or in soil can lead to mild to severe infections, which present with cellulitis, myonecrosis, and ecthyma gangrenosum. *Aeromonas* septicemia is strongly associated with immunocompromising conditions such as hematological malignancies and serious hepatobiliary disease. Among the various *Aeromonas* species, *Aeromonas hydrophila* (*A. hydrophila*) is the most commonly identified pathogen [1, 2]. Cirrhotic patients are immunocompromised and, hence, exhibit significant bacterial infection-associated morbidity and mortality rates (approximately 30%) [3]. When a patient develops septic shock, early goal-directed therapy and effective antibiotic treatment should be provided within the first hour of documented hypotension to increase their chances of survival [4, 5]. We describe a case of septic shock due to *A. hydrophila* bacteremia in a cirrhotic patient, which was successfully treated with immediate antimicrobial administration and multiple supportive therapies including endotoxin absorption and cytokine-absorbing hemofiltration.

## Case presentation

**Figure 1:**
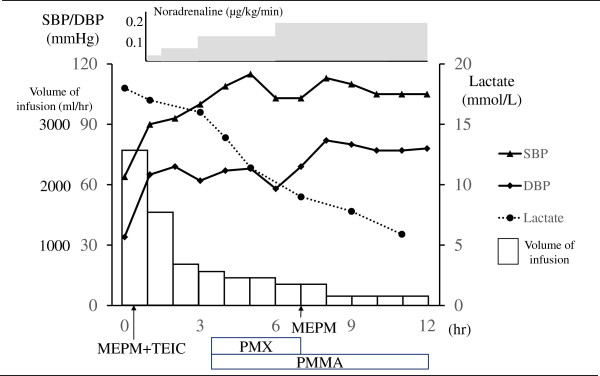
**Patient’s clinical course during the first 12 hours after his arrival.** DBP, diastolic blood pressure; MEPM, meropenem; PMMA, polymethylmethacrylate; PMX, polymyxin B hemoperfusion; SBP, systolic blood pressure; TEIC, teicoplanin.

**Figure 2:**
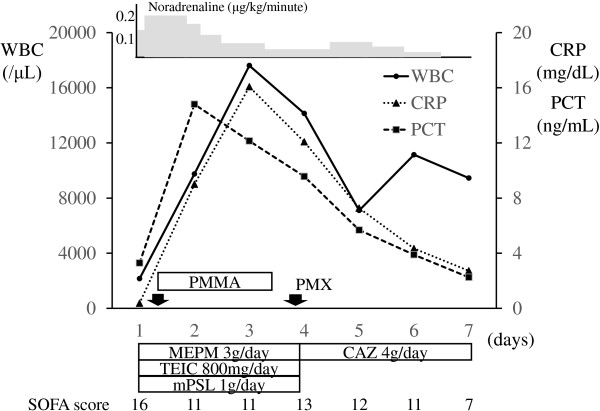
**The patient’s clinical course over the first 7 days after his admission.** CAZ, ceftazidime; CRP, C-reactive protein; MEPM, meropenem; mPSL, methylprednisolone; PCT, procalcitonin; PMMA, polymethylmethacrylate; PMX, polymyxin B hemoperfusion; SOFA, Sequential Organ Failure Assessment; TEIC, teicoplanin; WBC, white blood cell count.

A 71-year-old Japanese man complaining of gait difficulties and fever was brought to our emergency and critical care medical center by ambulance. He had had general fatigue for several days before admission. He had alcoholic liver cirrhosis (LC), which had been treated with diuretics and branched-chain amino acids for the past 3 months. He had been taking approximately 100g of alcohol per day for more than 30 years with a balanced diet. At 1 month before admission, his laboratory data were as follows: total bilirubin, 1.36mg/dL; albumin, 3.5g/dL; and international normalized ratio, 1.05. In addition, he had mild ascites, which was medically controlled. These findings were indicative of Child-Pugh class B LC. A gastrointestinal endoscopy had been performed the previous month, but it did not detect any esophageal or gastric varices. In addition, he had not had any episodes of hematemesis or produced bloody stools. On arrival, he appeared to be disturbed and distressed and was classified as 13 (E3V4M6) on the Glasgow Coma Scale. His vital signs were as follows: respiratory rate, 30 breaths/minute; pulse rate, 147 beats/minute and regular; blood pressure, 64/34mmHg; and temperature, 38.4°C. He did not have a headache; sore throat; cough; sputum; or chest, abdominal, or back pain. Auscultation of his lungs and heart produced normal findings. His abdomen was soft and flat and non-tender, and no skin rashes or infectious wounds were present on his extremities. Arterial blood gas analysis detected severe lactic acidosis (lactate concentration: 18mmol/L), which was indicative of septic shock (Table 1). Rapid fluid resuscitation with adequate doses of vitamin B administration was started immediately followed by tracheal intubation and mechanical ventilation according to the goal-oriented therapy protocol. Empirical antimicrobial treatment with meropenem and teicoplanin was administered 20 minutes after his arrival, and two sets of blood cultures were obtained at the same time. His laboratory data were indicative of multiple organ failure; that is, pancytopenia, coagulopathy, and acute kidney injury were detected together with high levels of procalcitonin and endotoxins (Table 1). However, a whole body computed tomography scan did not identify an infection focus. On admission to the emergency intensive care unit (EICU), his Acute Physiology and Chronic Health Evaluation (APACHE II) score was 32, and his Sequential Organ Failure Assessment (SOFA) score was 16. Because he exhibited persistent catecholamine-resistant hypotension, endotoxin absorption therapy based on polymyxin B hemoperfusion (PMX) and cytokine-absorption therapy involving the use of a polymethylmethacrylate (PMMA) membrane hemofilter were performed to counteract his multiple organ failure. Other supportive treatments for his refractory shock such as the infusion of immunoglobulins, methylprednisolone, and recombinant human soluble thrombomodulin (rhTM) were also initiated. The level of fluid resuscitation was adjusted to maintain his urinary output at 0.5 to 1.0mL/kg/hour (Figure 1), and he was transfused with 6, 14, and 30 units of red cell concentrate, fresh frozen plasma, and platelet concentrate, respectively, within the first 24 hours after his admission. The initial resuscitative treatments resulted in a reduction in his lactate and endotoxin level and the maintenance of his mean blood pressure above 65mmHg. Ongoing aggressive critical care led to a gradual tapering of the catecholamine dose and an improvement in his SOFA score (Figure 2). An examination of his blood culture detected *A. hydrophila*, so, based on the results of sensitivity tests, we replaced the meropenem and teicoplanin with ceftazidime. We finally diagnosed him with spontaneous *A. hydrophila* septicemia combined with refractory shock facilitated by an immunosuppressive condition (LC). On day 10, he was successfully extubated, and he was discharged from the EICU the next day. Antibiotics were administered for 2 weeks. The patient was transferred to a satellite hospital to continue his rehabilitation on day 19.

**Table 1:** **The patient’s arterial blood gas analysis and data findings on admission**

Arterial blood gas analysis (room air)		Complete blood cell count		Biochemistry	
pH	7.196	WBC	2150/μL	TP	5.2g/dL
PaCO_2_	21.9mmHg	Hgb	6.8g/dL	Albumin	2.5g/dL
PaO_2_	91.2mmHg	Hct	21.5%	AST	180U/L
HCO_3_	8.2mmol/L	PLT	2.7×10^4^/μL	ALT	70U/L
Base excess	–18.5mmol/L			T-Bil	1.60mg/dL
Lactate	18mmol/L			D-Bil	0.98mg/dL
Glucose	91mg/dL			BUN	22.8mg/dL
		**Coagulation**		Creatinine	1.68mg/dL
		PT	29%	Na	142mmol/L
		INR	1.84	K	3.6mmol/L
		APTT	89.9 seconds	Cl	106mmol/L
		Fibrinogen	<50mg/dL	CRP	0.37mg/dL
		D-dimer	6.6μg/mL	Procalcitonin	3.290ng/mL
		AT3	20%	Endotoxin	14.2pg/mL

ALT, alanine transaminase; APTT, activated partial thromboplastin time; AST, aspartate transaminase; AT3, antithrombin 3; BUN, blood urea nitrogen; Cl, chlorine; CRP, C-reactive protein; D-Bil, direct bilirubin; HCO_3_, bicarbonate; Hct, hematocrit; Hgb, hemoglobin; INR, international normalized ratio; K, potassium; Na, sodium; PaCO_2_, carbon dioxide partial pressure arterial; PaO_2_, oxygen partial pressure arterial; PLT, platelet count; PT, prothrombin time; T-Bil, total bilirubin; TP, total protein; WBC, white blood cell count.

## Discussion

The mortality rate of severe sepsis with or without shock in the intensive care setting is very high (about 23%) [6], and evidence-based recommendations have been published with the aim of improving the outcomes of severe sepsis and septic shock in critically ill patients [4, 7]. Severe sepsis and septic shock are associated with significantly higher mortality in cirrhotic patients than in non-cirrhotic patients [8]. The most common bacterial infections seen in cirrhotic patients are spontaneous bacterial peritonitis (25 to 31%), urinary tract infections (20 to 25%), pneumonia (15 to 21%), bacteremia (12%), and soft tissue infections (11%), and the most common causative organisms of such infections are *Staphylococcus* species, *Enterococcus* species, and methicillin-resistant *Staphylococcus aureus* among Gram-positive bacteria and *Escherichia coli*, *Klebsiella* species, and *Enterobacter* species among Gram-negative bacteria. Other bacterial infections such as those involving *Vibrio* species, *Aeromonas* species, *Clostridium* species, and *Mycobacterium tuberculosis* are more virulent [3, 9]. *Aeromonas* species cause a variety of diseases including acute gastroenteritis, skin and soft tissue infections, and septicemia. *Aeromonas* septicemia is strongly associated with immunosuppressive conditions such as cirrhosis and hematological malignancies. Among the various *Aeromonas* species, *A. hydrophila* is the most commonly identified pathogen [1, 2, 10]. Bacterial translocation is associated with the pathogenesis of spontaneous bacterial peritonitis and spontaneous bacteremia in cirrhotic patients. In the present case, as the patient had no trauma episodes or wound site, oral transmission was the probable cause of infection pathway. The intravenous administration of carbapenems or third generation cephalosporins combined with aminoglycosides or fluoroquinolones is recommended as an empiric treatment for serious bacterial infections in cirrhotic patients [3]. In the present case, the early recognition of septic shock and the initiation of rapid resuscitation techniques including the administration of antimicrobials were considered to be the most important factors in the saving of our patient’s life. It is reported that delaying antibiotic treatment until after the identification of shock is associated with a worse prognosis [11]. Various kinds of treatment have been used to treat severe sepsis and septic shock. For instance, direct hemoperfusion with PMX is suggested to have favorable effects on blood pressure, oxygenation, and mortality [12], and hemoperfusion using PMMA membrane hemofilters, which have an enhanced ability to absorb cytokines, might improve the mortality rate of such conditions [13]. In addition, the early infusion of stress dose steroids should be considered in patients with septic shock whose blood pressure issues are poorly responsive to fluid resuscitation and catecholamine [7], and the administration of rhTM might be effective at treating sepsis-induced coagulopathy [14]. Although further investigations are required to confirm whether such supportive therapies are effective, aggressive treatment with multiple modalities and combined therapies could be effective in patients with severe septic shock who are unresponsive to conventional management strategies.

## Conclusions

We described a case of septic shock due to *A. hydrophila* bacteremia in a patient with LC. In addition to the early identification of septic shock and the use of rapid resuscitation techniques including the administration of antimicrobials, aggressive multidisciplinary intensive care resulted in the successful treatment of our patient’s septic shock.

## Consent

Written informed consent was obtained from the patient for publication of this case report and accompanying images. A copy of the written consent is available for review by the Editor-in-Chief of this journal.

## Abbreviations

EICU: Emergency intensive care unit
LC: Liver cirrhosis
PMMA: Polymethylmethacrylate
PMX: Polymyxin B hemoperfusion
rhTM: Recombinant human soluble thrombomodulin
SOFA: Sequential Organ Failure Assessment.

## References

[1] Parker JL, Shaw JG: ***Aeromonas*****spp. clinical microbiology and disease.** *J Infect* 2011, **62:**109–118. 10.1016/j.jinf.2010.12.003 10.1016/j.jinf.2010.12.003 21163298

[2] Igbinosa IH, Igumbor EU, Aghdasi F, Tom M, Okoh AI: **Emerging*****Aeromonas*****species infections and their significance in public health.** *Sci World J* 2012, **2012:**625023. 10.1100/2012/625023 PMC3373137 22701365

[3] Chalermrat B, Disaya C: **Bacterial infections other than spontaneous bacterial peritonitis in cirrhosis.** *World J Hepatol* 2012, **4:**158–168. 10.4254/wjh.v4.i5.158 10.4254/wjh.v4.i5.158 PMC3365435 22662285

[4] Rivers E, Nguyen B, Havstad S, Ressler J, Muzzin A, Knoblich B, Peterson E, Tomlanovich M, Early Goal-Directed Therapy Collaborative Group: **Early goal-directed therapy in the treatment of severe sepsis and septic shock.** *N Engl J Med* 2001, **345:**1368–1377. 10.1056/NEJMoa010307 10.1056/NEJMoa010307 11794169

[5] Kumar A, Roberts D, Wood KE, Light B, Parrillo JE, Sharma S, Suppes R, Feinstein D, Zanotti S, Taiberg L, Gurka D, Kumar A, Cheang M: **Duration of hypotension before initiation of effective antimicrobial therapy is the critical determinant of survival in human septic shock.** *Crit Care Med* 2006, **34:**1589–1596. 10.1097/01.CCM.0000217961.75225.E9 10.1097/01.CCM.0000217961.75225.E9 16625125

[6] Ogura H, Gando S, Saitoh D, Takeyama N, Kushimoto S, Fujishima S, Mayumi T, Araki T, Ikeda H, Kotani J, Miki Y, Shiraishi S, Suzuki K, Suzuki Y, Takuma K, Tsuruta R, Yamaguchi N, Aikawa N, Japanese Association for Acute Medicine Sepsis Registry (JAAMSR) Study Group: **Epidemiology of severe sepsis in Japanese intensive care units: a prospective multicenter study.** *J Infect Chemother* 2014,**20**(3)**:**157–162. 10.1016/j.jiac.2013.07.006 10.1016/j.jiac.2013.07.006 24530102

[7] Dellinger RP, Levy MM, Carlet JM, Bion J, Parker MM, Jaeschke R, Reinhart K, Angus DC, Brun-Buisson C, Beale R, Calandra T, Dhainaut JF, Gerlach H, Harvey M, Marini JJ, Marshall J, Ranieri M, Ramsay G, Sevransky J, Thompson BT, Townsend S, Vender JS, Zimmerman JL, Vincent JL, International Surviving Sepsis Campaign Guidelines Committee; American Association of Critical-Care Nurses; American College of Chest Physicians; American College of Emergency Physicians; Canadian Critical Care Society; European Society of Clinical Microbiology and Infectious Diseases; European Society of Intensive Care Medicine; European Respiratory Society; International Sepsis Forum; Japanese Association for Acute Medicine; Japanese Society of Intensive Care Medicine; Society of Critical Care Medicine; Society of Hospital Medicine; Surgical Infection Society; World Federation of Societies of Intensive and Critical Care Medicine: Surviving Sepsis Campaign: **International guideline for management of severe sepsis and septic shock: 2008.** *Crit Care Med* 2008, **36:**296–327. 10.1097/01.CCM.0000298158.12101.41 10.1097/01.CCM.0000298158.12101.41 18158437

[8] Gustot T, Felleiter P, Pickkers P, Sakr Y, Rello J, Velissaris D, Pierrakos C, Taccone FS, Sevcik P, Moreno C, Vincent JL, the EPIC II Group of Investigators: **Impact of infection on the prognosis of critically ill cirrhotic patients: results from a large worldwide study.** *Liver Int* 2014,**10**(1111)**:**12520. 10.1111/liv.12520 24606193

[9] Shizuma T, Tanaka C, Mori H, Fukuyama N: **Investigation of bacteremia due to*****Aeromonas*****species and comparison with that due to enterobacteria in patients with liver cirrhosis.** *Gastroenterol Res Pract.* 2011, **2011:**930826. 10.1155/2011/930826 PMC3255164 22253618

[10] Choi JP, Lee SO, Kwon HH, Kwak YG, Choi SH, Lim SK, Kim MN, Jeong JY, Choi SH, Woo JH, Kim YS: **Clinical significance of spontaneous*****Aeromonas*****bacterial peritonitis in cirrhotic patients: a matched case-control study.** *Clin Infect Dis* 2008, **47:**66–72. 10.1086/588665 10.1086/588665 18484880

[11] Puskarich MA, Trzeciak S, Shapiro NI, Arnold RC, Horton JM, Studnek JR, Kline JA, Jones AE, Emergency Medicine Shock Research Network (EMSHOCKNET): **Association between timing of antibiotic administration and mortality from septic shock in patients treated with a quantitative resuscitation protocol.** *Crit Care Med* 2011, **39:**2066–2071. 10.1097/CCM.0b013e31821e87ab 10.1097/CCM.0b013e31821e87ab PMC3158284 21572327

[12] Cruz DN, Perazella MA, Bellomo R, de Cal M, Polanco N, Corradi V, Lentini P, Nalesso F, Ueno T, Ranieri VM, Ronco C: **Effectiveness of polymyxin B-immobilized fiber column in sepsis: a systematic review.** *Crit Care* 2007, **22:**R47. 10.1186/cc5780 PMC2206475 17448226

[13] Oda S, Sadahiro T, Hirayama Y, Nakamura M, Watanabe E, Tateishi Y, Hirasawa H: **Non-renal indications for continuous renal replacement therapy: current status in Japan.** *Contrib Nephrol* 2010, **166:**47–53. 10.1159/000314851 20472991

[14] Yamakawa K, Fujimi S, Mohri T, Matsuda H, Nakamori Y, Hirose T, Tasaki O, Ogura H, Kuwagata Y, Hamasaki T, Shimazu T: **Treatment of effects of recombinant human soluble thrombomodulin in patients with severe sepsis: a historical control study.** *Crit Care* 2011, **15:**R123. 10.1186/cc10228 10.1186/cc10228 PMC3218981 21569368

